# Role of SUMO-Specific Protease 2 in Reprogramming Cellular Glucose Metabolism

**DOI:** 10.1371/journal.pone.0063965

**Published:** 2013-05-14

**Authors:** Shuang Tang, Gang Huang, Xuemei Tong, Lian Xu, Rong Cai, Jie Li, Xiang Zhou, Shaoli Song, Chen Huang, Jinke Cheng

**Affiliations:** 1 Department of Nuclear Medicine, Renji Hospital, Shanghai Jiao Tong University, School of Medicine, Shanghai, China; 2 Institute of Health Sciences, Shanghai Jiao Tong University School of Medicine (SJTUSM) and Shanghai Institutes for Biological Sciences (SIBS), Chinese Academy of Sciences (CAS), Shanghai, China; 3 Department of Biochemistry and Molecular Cell Biology, Shanghai Key Laboratory for Tumor Microenviroment and Inflammation, Shanghai Jiao Tong University School of Medicine, Shanghai, China; University of South Alabama, United States of America

## Abstract

Most cancer cells exhibit a shift in glucose metabolic strategy, displaying increased glycolysis even with adequate oxygen supply. SUMO-specific proteases (SENPs) de-SUMOylate substrates including HIF1α and p53,two key regulators in cancer glucose metabolism, to regulate their activity, stability and subcellular localization. However, the role of SENPs in tumor glucose metabolism remains unclear. Here we report that SUMO-specific protease 2 (SENP2) negatively regulates aerobic glycolysis in MCF7 and MEF cells. Over-expression of SENP2 reduces the glucose uptake and lactate production, increasing the cellular ATP levels in MCF7 cells, while SENP2 knockout MEF cells show increased glucose uptake and lactate production along with the decreased ATP levels. Consistently, the MCF7 cells over-expressing SENP2 exhibit decreased expression levels of key glycolytic enzymes and an increased rate of glucose oxidation compared with control MCF7 cells, indicating inhibited glycolysis but enhanced oxidative mitochondrial respiration. Moreover, SENP2 over-expressing MCF7 cells demonstrated a reduced amount of phosphorylated AKT, whereas SENP2 knockout MEFs exhibit increased levels of phosphorylated AKT. Furthermore, inhibiting AKT phosphorylation by LY294002 rescued the phenotype induced by SENP2 deficiency in MEFs. In conclusion, SENP2 represses glycolysis and shifts glucose metabolic strategy, in part through inhibition of AKT phosphorylation. Our study reveals a novel function of SENP2 in regulating glucose metabolism.

## Introduction

Small ubiquitin-like modifier (SUMO) mediates a diverse array of cellular events by conjugating to numerous protein substrates, regulating the activity, stability, and subcellular localization of modified proteins. SUMO conjugation is a dynamic and reversible process, which can be readily reversed by a family of Sentrin/SUMO-specific proteases SENPs [1], [2]. The SENP family involves six members in human, SENP1-3 and SENP5-7, and each has different cellular location, substrate specificity and biological function. Although SENPs are known to reverse SUMOylation in many different systems, their physiological roles have not been precisely defined [3].

Aerobic glycolysis or Warburg effect is considered as a hallmark of most cancer cells [4]. Compared with oxidative mitochondrial respiration, aerobic glycolysis is an inefficient way of glucose catabolism in terms of ATP production. To ensure adequate energy for fast proliferation, tumor cells have to take up excessive glucose. This feature has been used to sensitively image cancer in clinics with the glucose (18F)-ﬂuoro-2-deoxy-D-glucose (FDG) through the positron emission tomography (PET) [4]. Although the “Warburg effect” has been widely observed in a variety of cancer cells, the underlying mechanisms are still not fully understood.

Several studies have indicated that SENPs may be essential for cancer glycolysis. For example, SENP1 is essential for stabilization of HIF1α during hypoxia [3]. SENP2-dependent regulation of Mdm2 is sensitive to its p53-binding activity [5]. HIF1α and P53 are both very important regulators of cancer glycolysis. These studies raise the possibility that SENPs play a role in glucose metabolism in cancer cells. The purpose of our work is to investigate the function of SENP2 in glucose metabolism.

Here we report that SENP2 negatively regulates aerobic glycolysis. Over-expression of SENP2 in MCF7 breast cancer cells reduces the glucose uptake and lactate production through repression of mRNA levels of key glycolytic enzymes, while SENP2 knockout MEF cells display increased glucose uptake and lactate production with elevated mRNA levels of key glycolytic enzymes compared to WT MEF cells. Moreover, SENP2 over-expressed MCF7 cells show reduced glycolysis but increased ATP levels and glucose oxidation. Therefore, SENP2 may play a role in reprogramming glucose metabolism from aerobic glycolysis to TCA cycle. Mechanism study indicates that AKT phosphorylation (Ser473) is involved in this process. Taken together, SENP2 plays a negative role in glucose metabolism, most likely by regulating AKT phosphorylation.

## Materials and Methods

### 1. Cell Culture

Human breast cancer cell line MCF7 is gifted from the Shanghai key laboratory for tumor microenviroment and inflammation. SENP2 MEF cells were isolated from E10.5 embryos as previously described [3], [6]. These cells were incubated in Dulbecco’s modified Eagle’s medium (DMEM, Gibco) with 10% fetal bovine serum (FBS, HyClone) at 37°C.

### 2. RNA Interference

Plasmid pbabe-SENP2 and pbabe-vector were generated using standard cloning procedures. The retrovirus containing pbabe-SENP2 or pbabe-vector was transfected into MCF7 cells to generate MCF7-SENP2 and MCF7-CON cells. These cell lines were cultured in DMEM with 10% FBS and 3 µg/mL puromycin.

### 3. Real-time Quantitative PCR

Real-time PCR was performed following the previously published protocol reported (11). Fluorescence real-time RT-PCR was performed with the double-stranded DNA dye SYBR Green PCR Core Reagents (PE Biosystems) using the ABI PRISM 7300 system (Perkin–Elmer). All data were analyzed using ABI PRISM SDS 2.0 software (Perkin–Elmer). Pairs of PCR primers used to amplify target genes were shown as Table S1.

### 4. Western Blotting

Protein extracts were equally loaded onto 10% SDS polyacrylamide gels, electrophoresed, and transferred to nitro cellulose membranes (Amersham Bioscience). After blocking with 5% non-fat milk in PBS, the membranes were probed with antibodies against HK2, AKT, pAKT (the 473 site), P21, β-actin (Cell Signaling), P53 (Santa Cruz Biotech), MYC (Beyotime) and HIF1a (BD Biosciences), followed by horseradish peroxidase-conjugated secondary antibodies. The signals were detected by chemiluminescent substrate kit (Millipore Corporation). Image J software was used to quantify protein levels.

### 5. Immunohistochemistry

Paraffin-embedded tissues of 30 cases of breast tumor patients were obtained from the Department of Pathology, Renji Hospital, Shanghai Jiao Tong University School of Medicine,including 24 infiltrating ductal carcinoma of breast and 6 breast adenofibroma samples. The immunohistochemical (IHC) analysis was performed on the 4 um thick fraction mounted on charged slides and sectioned from each clinical sample. Tissue slides were immunohistochemically stained by antibody against SENP2 (Gene Tex) and Glucose transporter 1 (Glut1) (Millipore), then visualized by standard avidin–biotinylated peroxidase complex method. Hematoxylin was used for counterstaining and morphologic images were observed with Olympus BX51 microscope [7].

IHC grading was defined based on intensity and frequency derived from the staining results. The staining intensity was scored as negative (0), weak (+1), moderate (+2), or strong (+3). The frequency of positive cells in a section was scored as negative (0), less than 25% (+1), 25–50% (+2), 51–75% (+3), or more than 75% (+4). IHC grading was assigned by multiplying the intensity score by the frequency score, as follows: −, absent expression (0); +, weak expression (1–4), ++, moderate expression (5–8); +++, high expression (9–12) [8]. Absent and weak expression was considered as negative expression, while moderate and high expression was considered as positive expression.

### 6. Glucose Uptake and Lactate Production Measurements

3×10^5^ MCF7 cells or 1×10^5^ MEF cells were seeded in 6-well plates and cultured in DMEM medium for 72 hours or 48 hours. Glucose uptake was measured using the glucose assay kit (Jiancheng) and lactate production was measured using the Lactate assay kit (CMA, Microdialysis) per the manufacturer’s protocol. Cell numbers at the end of experiments were calculated to normalize the glucose uptake and the lactate production results.

### 7. ^14^CO_2_ Release Assay

*^14^CO_2_* released from glucose oxidation was determined by incubating cells in bicarbonate free medium containing 1 µCi/mL [6-^14^C] glucose for 3 h and testing radioactivity of collected *^14^CO_2_*. This method is designed according to the principle and protocol reported previously [9], [10]. phenylethylamine was used to absorb *CO_2_*. *^14^CO_2_* resulting from oxidized glucose was quantified by scintillation counting of the phenylethylamine. Each experiment was performed in triplicate and the results were normalized to total protein.

### 8. ATP Measurement

To measure cellular ATP levels, an ATP Bioluminescence Assay Kit (Roche) was used according to manufacturers’ instructions. The intracellular ATP levels were determined using a luciferin-luciferase assay with luminescence being measured by Infinite 200 PRO Multimode Microplate Reader (TECAN,). The ATP level results were cell number normalized.

### 9. Statistical Analysis

Graphpad prism software was used for statistical analysis and for plotting graphs. All data is presented as mean ± SEM of three independent experiments. Statistical significance was evaluated by two-tailed paired Student’s t-test. Spearman rank correlation analysis was performed to analyze the relevance between Glut1 and SENP2 protein expression. Statistical significance was set at P<0.05.

### 10. Ethics Statement

This study is approved by the Ethics Committee of Renji Hospital, Shanghai Jiao Tong University School of Medicine. This study was performed in strict accordance with the recommendations in the Guide for the Care and Use of Human Samples from Renji Hospital. We declare no ethical or conflicts of interest.

In our study, oral informed consent was obtained. Because the Paraffin-embedded tissue samples from patients with cancer were produced in 2002 by the Department of Pathology of Renji Hosptal, Shanghai Jiao Tong University School of Medicine, all the patients have left hospital and some of them have passed away. We can only get the oral informed consent from the patients or the family members of the patient who has passed away by telephone. But when the Department of Pathology of Renji hospital made the original human tissue samples in 2002, written informed consent was confirmed by every patient before surgery.

## Results

### 1. SENP2 is Expressed at Lower Levels in Breast Cancer Cells and Over-expression of SENP2 Reduces Glucose Uptake in MCF7 Cells

**Figure 1:**
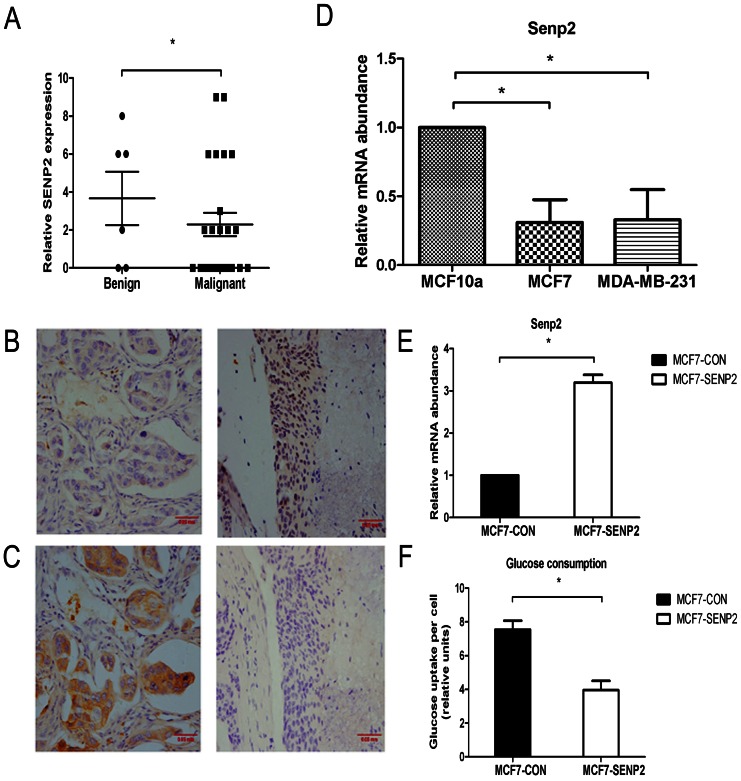
SENP2 is weakly expressed in breast cancer cells and over-expression of SENP2 reduces glucose uptake in MCF7 cells. (A) Expression of SENP2 protein in benign breast adenofibroma (n = 6) and malignant breast cancer tissue (n = 24) analyzed by IHC with anti-SENP2 antibody. (B) Representative IHC results staining with anti-SENP2 and (C) anti-GLUT1 antibodies. (D) Expression of SENP2 mRNA in MCF10a, MCF7 and MDA-MB231 cells analyzed by Q-PCR. (E) Higher expression of SENP2 mRNA in MCF7-SENP2 cells than MCF7-CON cells analyzed by Q-PCR. (F) Glucose uptake results of MCF7-CON and MCF7-SENP2 cells. The data were normalized by cell number. *P<0.05.

To elucidate the possible role of SENP2 in cancer cell metabolism, we first analyzed the expression levels of this factor in human breast cancer tissues. The protein levels of SENP2 protein were significantly lower in breast cancer tissue than benign breast adenofibroma tissue from patients (Fig. 1.A). Furthermore, the mRNA levels of SENP2 were significant reduced in MCF7 and MDA-MB-231 cells, two breast cancer cell lines, when compared to normal breast cell line MCF10a (Fig. 1.D). Interestingly, the expression levels of SENP2 were negatively correlated with the levels of glucose transporter 1 (GLUT1), a key player in breast cancer cell glucose metabolism, in 30 human breast tumor samples (Fig. 1.B, C, Table 1,) and Spearman Rank-order Coefficient (*r*s) was −0.42691, P<0.05 ), suggesting an inhibitory role of SENP2 in breast cancer glucose uptake.

**Table 1:** Negative correlation was found between SENP2 and GLUT1 protein in 30 breast tumor tissues.

	SENP2 positive	SENP2 negative	Sum up
GLUT1 positive	3	16	19
GLUT1 negative	6	5	11
Sum up	9	21	

Spearman Correlation Coefficients, N = 30. Spearman rs = −0.42691. P = 0.0186<0.05.

To test this possibility, we stably over-expressed SENP2 in the MCF7 breast cancer cell line where the endogenous SENP2 levels were significantly reduced compared to non-cancer cell (Fig. 1.D and E). As expected, MCF7-SENP2 cells uptook significantly less amount of glucose than MCF7-CON cells (Fig. 1.F), indicating SENP2 represses glucose uptake in breast cancer cells.

### 2. SENP2 Over-expression in MCF7 Cells Reprograms Glucose Metabolism

**Figure 2:**
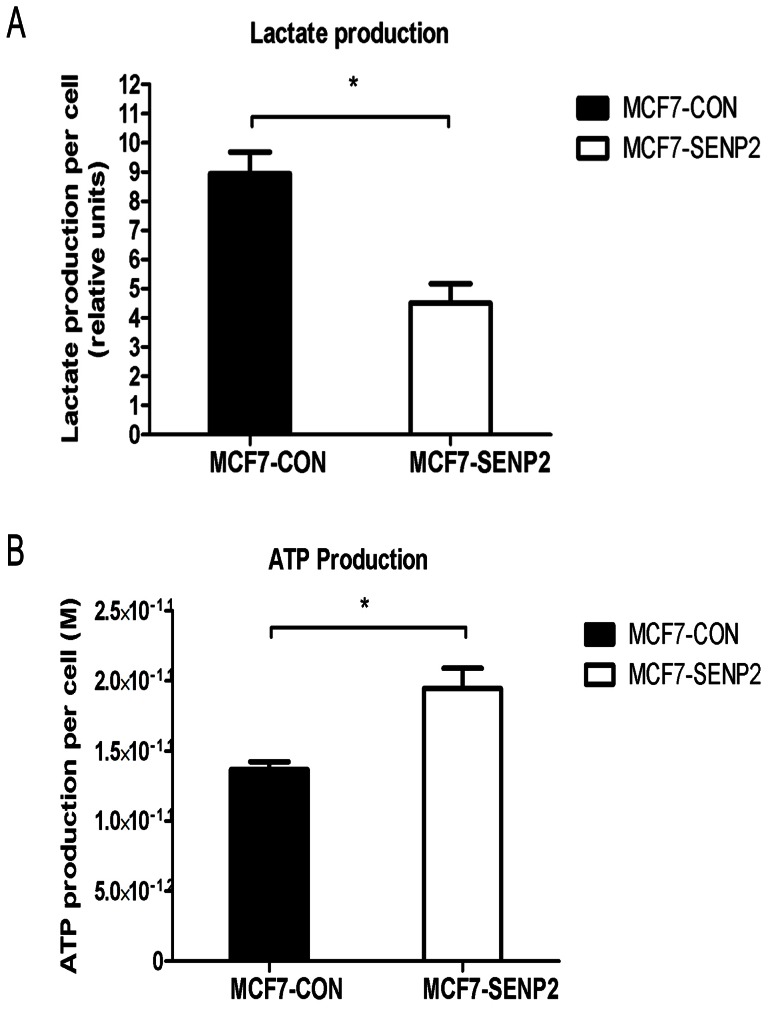
SENP2 over-expressing MCF7 cells display a shift of glucose metabolic strategy. (A) Lactate production and (B) ATP level in MCF7-CON and MCF7-SENP2 cells. The data were presented as the mean±SD of triplicate samples and normalized by cell number. *P<0.05.

Although the oxidative mitochondrial metabolism is a more efficient way to produce energy, most cancer cells rely on aerobic glycolysis for ATP production to accommodate their rapid proliferation. To test whether the decreased glucose consumption in the MCF7-SENP2 cells is due to repressed glycolysis, we compared lactate production between MCF7-CON and MCF7-SENP2 cells. As shown in Fig. 2.A, MCF7-SENP2 cells produced much less lactate than MCF7-CON cells (p<0.001), indicating SENP2 over-expression in MCF7 cells leads to reduced glycolysis. Consistently, MCF7-SENP2 cells produced more ATP compared to control cells (Fig. 2.B), suggesting that SENP2 over-expression results in switch from glycolysis to oxidative mitochondrial metabolism for ATP production.

### 3. SENP2 Reduces Key Glycolytic Enzymes Expression and Increases Glucose Oxidation

**Figure 3:**
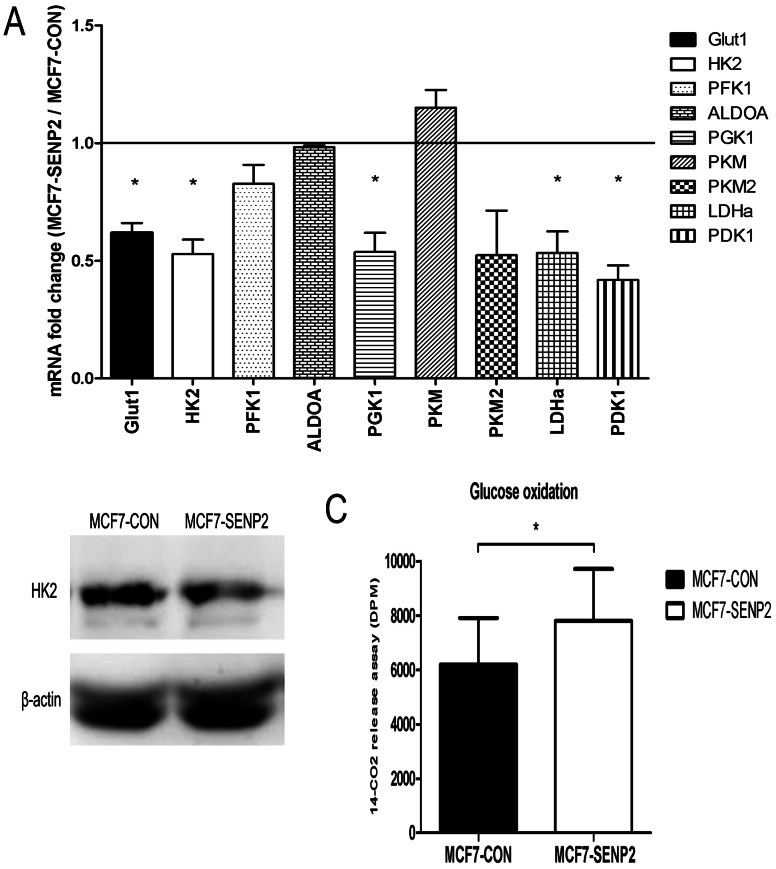
SENP2 reduces key glycolytic enzymes expression and increases glucose oxidation. (A) Fold Change of mRNA expression of Glut1 and key glycolytic enzymes in MCF7-SENP2 cells compared with MCF7-CON cells analyzed by Q-PCR. (B) Western blot analysis of HK2 in MCF7-CON and MCF7-SENP2 cells. (C) Glucose oxidation of MCF7-CON and MCF7-SENP2 cells analyzed by ^14^CO2 Release Assay. *P<0.05.

To explore the mechanism underlying SENP2 over-expression mediated less glucose consumption and lactate production, we compared mRNA levels of key glycolytic enzyme genes between MCF7-CON and MCF7-SENP2 cells. As shown in Fig. 3.A, the mRNA levels of most key glycolytic enzymes, including Glut1, hexokinase 2 (HK2), phosphofructokinase 1 (PFK1), phosphoglycerate kinase 1 (PGK1) and pyruvate kinase isozyme M2 (PKM2), were significantly decreased in MCF7-SENP2 cells compared with MCF7-CON cells. Western blotting confirmed that levels of HK2 protein were reduced in MCF7-SENP2 cells compared to MCF7-CON cells (Fig. 3.B). In addition, mRNA levels of of lactate dehydrogenase A (LDHA), the enzyme catalyzing lactate formation from pyruvate, were also significantly decreased in MCF7-SENP2 cells compared to MCF7-CON cells (Fig. 3.A).

Since the inhibited glycolysis induced by SENP2 over-expression does not accompany with reduced ATP level, it is possible that SENP2 induces a glucose metabolic switch from aerobic glycolysis to oxidative mitochondrial metabolism, which is a more efficient way to produce ATP. To test this hypothesis, we performed the glucose oxidation assay [11], [12] in control and MCF7-SENP2 cells. Control and MCF7-SENP2 cells were incubated with ^14^C labelled glucose and the release of ^14^CO_2_ from [6-^14^C] glucose were measured. As shown in Fig. 3.C, mean glucose oxidation rates were increased by approximately 25.93% in MCF7-SENP2 cells compared to MCF7-CON cells. This result confirmed that over-expression of SENP2 results in higher percentage of glucose switch mitochondrial oxidative phorphorylation to more efficiently produce ATP. Consistently, the mRNA levels of pyruvate dehydrogenase kinase 1 (PDK1), an enzyme that prevents the conversion of pyruvate to acetyl-CoA, were much lower in MCF7-SENP2 cells than MCF7-CON cells (Fig. 3.A). Taken together, our data suggest that SENP2 induces a glucose metabolic switch from aerobic glycolysis to oxidative mitochondrial respiration.

### 4. Knockout of SENP2 Leads to Increased Aerobic Glycolysis in MEF Cells

**Figure 4:**
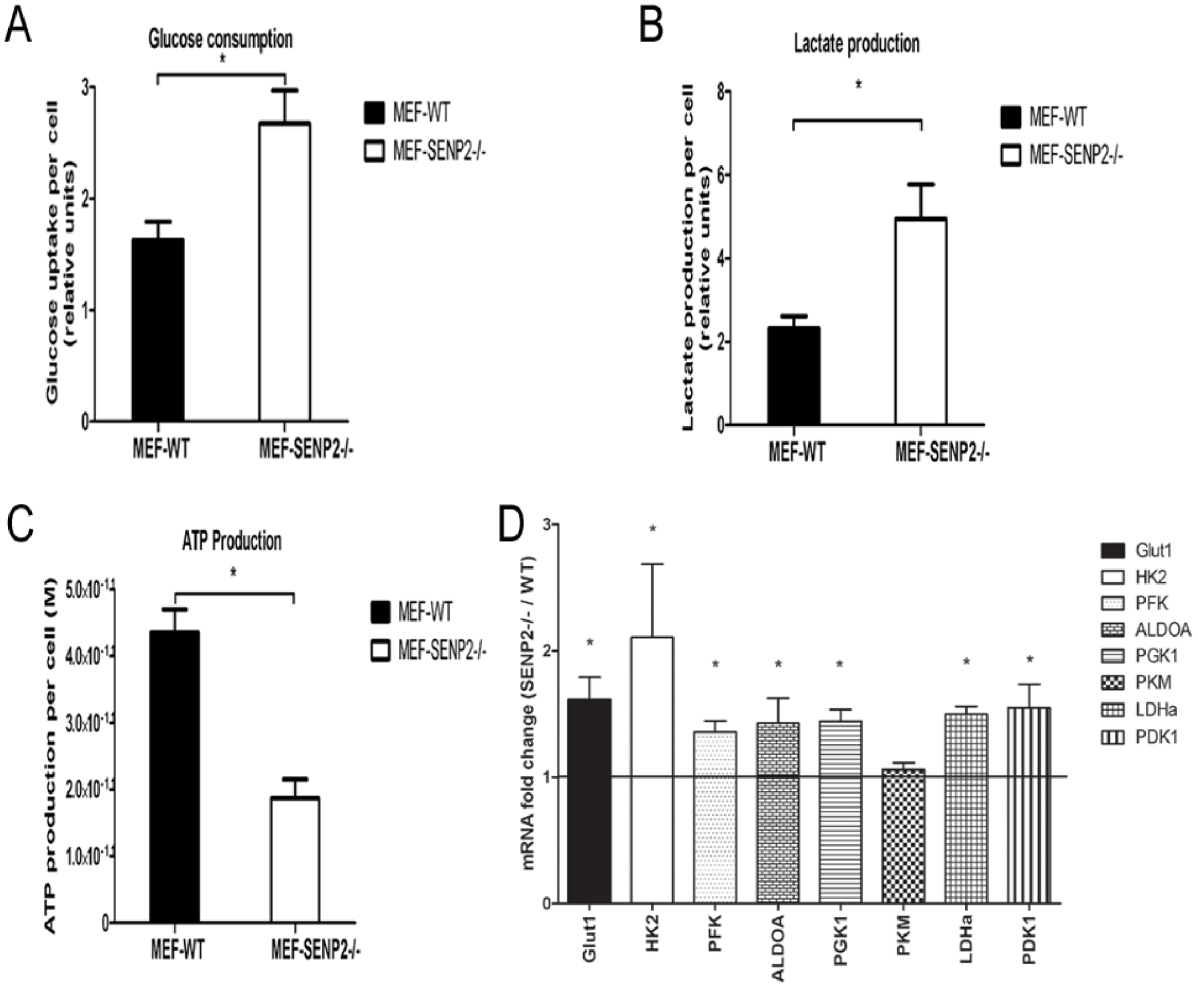
Knockout of SENP2 leads to increased aerobic glycolysis in MEF cells. (A) Glucose uptake, (B) Lactate production and (C) ATP level in WT and SENP2−/− MEF cells. (D) Fold Change of mRNA expression of Glut1 and key glycolytic enzymes in SENP2−/− MEF cells compared with WT MEF cells analyzed by Q-PCR. All the data were presented as the mean±SD of triplicate samples and normalized by cell number. *P<0.05.

To further confirm that SENP2 indeed represses aerobic glycolysis, we carried out glucose uptake and lactate assays on immortalized SENP2−/− and WT MEFs. SENP2−/− MEFs consistently uptook significantly higher amounts of glucose and produced more lactate than WT cells (Fig. 4.A, B). Moreover, SENP2−/− MEFs displayed significantly reduced levels of ATP compared to WT cells (Fig. 4.C). In line with these observations, the mRNA levels of most key glycolytic enzymes were increased in SENP2−/− MEF cells compared to WT cells (Fig. 4.D). All together, consistent with the results that over-expression of SENP2 reduces glycolysis in MCF7 cells, SENP2 deficiency in MEFs increases aerobic glycolysis, resulting in increased lactate production and reduced ATP levels.

### 5. Reduced AKT Phosphorylation Contributes to SENP2-mediated Repression of Glycolysis

**Figure 5:**
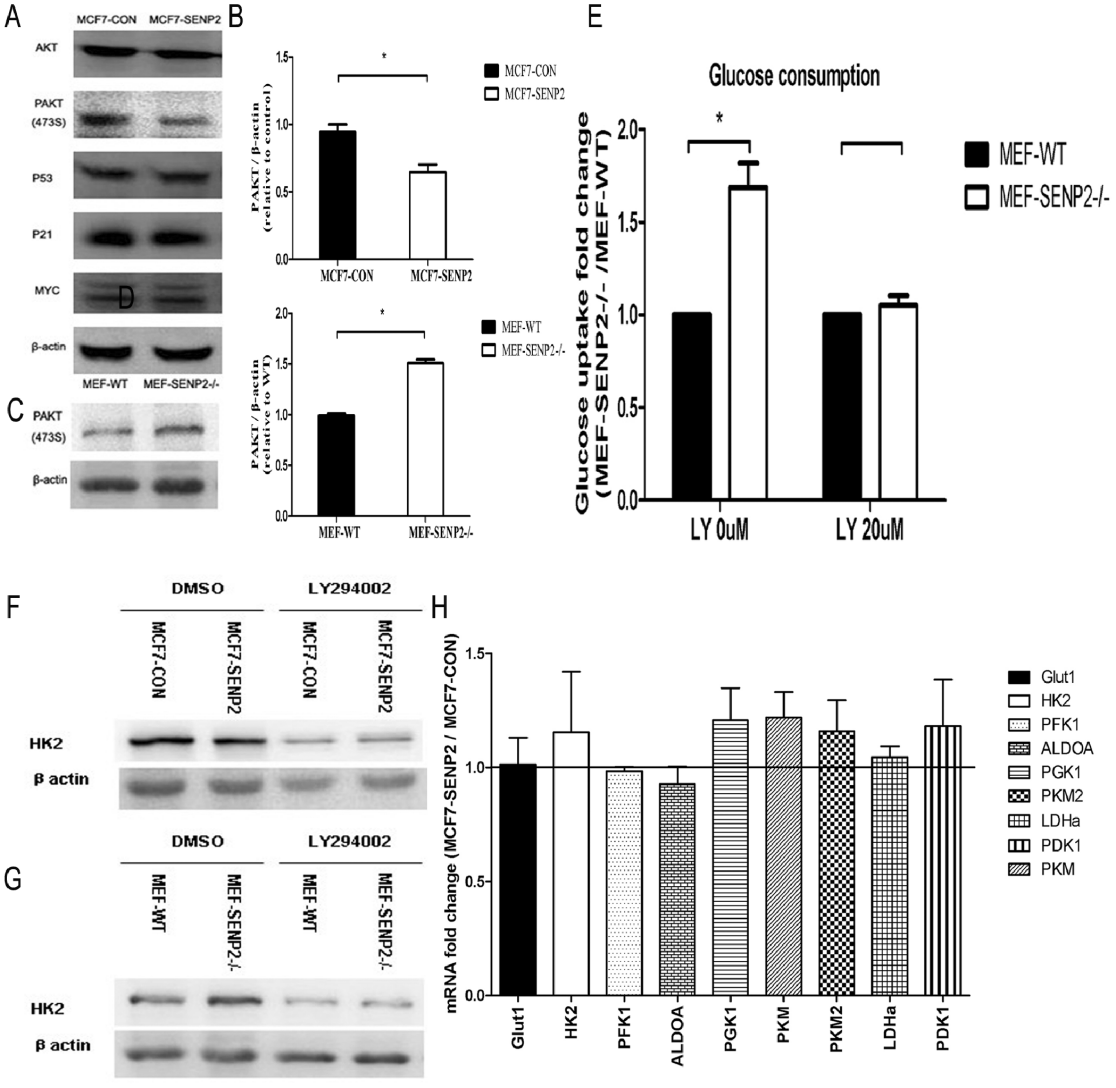
AKT phosphorylation is involved in glucose metabolism regulated by SENP2 in MCF7 cells. (A) Western blotting of phosphorylated AKT (473S), P53, P21 and MYC in MCF7-CON and MCF7-SENP2 cells. (B) Quantification of PAKT protein levels shown on figure (A) after normalization to β-actin protein. (C) Western blotting of phosphorylated AKT (473S) in MEF-WT and MEF-SENP2−/− cells. (D) Quantification of PAKT protein levels shown on figure (C) after normalization to β-actin protein. (E) Fold change of glucose uptake after 0 uM and 20 uM LY294002 treatment for 48 h between MEF-SENP2−/− and MEF-WT cells. (F) Western blotting of HK2 in of MCF7-CON and MCF7-SENP2 cells after incubating with DMSO or LY294002 for 48 h. (G) Western blotting of HK2 in of MEF-WT and MEF-SENP2−/− cells after incubating with DMSO or LY294002 for 48 h. (H) After LY294002 treatment for 48 h, fold Change of mRNA expression of Glut1 and key glycolytic enzymes in MCF7-SENP2 cells compared with MCF7-CON cells analyzed by Q-PCR.

To further dissect the molecular mechanisms underlying SENP2 induced inhibition of glycolysis, we analyzed a number of glycolytic regulators in MCF7 cells. Although total AKT, P53, P21 and MYC protein levels were normal in MCF7-SENP2 cells (Fig. 5.A) and our data suggested that HIF1a not be essential in SENP2-mediated repression of glycolysis (Fig. S1), phosphorylated AKT (473S) levels were significantly reduced in MCF7-SENP2 cells compared to MCF7-CON cells (Fig. 5.A, B). Consistently,phosphorylated AKT (473S) levels were significantly elevated in SENP2−/− MEF cells compared with WT MEF cells (Fig. 5.C, D). To test whether SENP2 regulates glycolysis through modulation of the phosphorylation levels of AKT, we treated WT and SENP2−/− MEFs with LY294002, a PI3 kinase inhibitor that blocks AKT phosphorylation. We found that inhibition of AKT phosphorylation by LY294002 rescued the hyper-glucose uptake in the SENP2−/− MEFs compared to WT MEFs (Fig. 5.E, Fig. S2). Furthermore, the levels of HK2 protein, which were lower in MCF7-SENP2 than MCF7-CON, were comparable after LY294002 treatment in MCF7-SENP2 and MCF7-CON cells (Fig. 5.F). The LY294002 treatment also reduced the levels of HK2 protein in SENP2−/− MEFs down to those in WT MEFs (Fig. 5.G). Finally, the mRNA levels of key glycolytic enzyme genes in MCF7-CON and MCF7-SENP2 cells were comparable after LY294002 treatment (Fig. 5.H). Collectively, our findings indicate that phosphorylation of AKT is an essential element in the SENP2-mediated regulation of glucose metabolism.

## Discussion

In this study, we show that SENP2 negatively regulates glycolysis in MCF7 and MEF cells. SENP2 over-expression leads to significantly reduced glucose uptake and lactate production in MCF7 cells, while SENP2 knockout in MEFs results in increased glucose uptake and lactate production. We also discovered a negative correlation between SENP2 and GLUT1 in human breast tumor samples, which might indicate a role of SENP2 in regulating glucose metabolism *in vivo*. Although a few recent studies have suggest a role of SUMOylation in metabolism [13], [14], [15], For example, SUMO1 impairs glucose-stimulated insulin secretion by blunting the β-cell exocytotic response to Ca^(2+)^ [13]. SUMO1 negatively regulates reactive oxygen species production from NADPH oxidases [14]. Agbor et al reported that over-expression of SUMO-1 in mammalian cancer cells resulted in increased hypoxia-induced glycolysis [15]. Our study provides the direct evidence that SENP2 is an important regulator of glucose metabolism.

We also explored the mechanisms of SENP2 in regulating glucose metabolism. Our data demonstrate that phosphorylation of AKT (473S) is a key mediator in the regulation of glucose metabolism by SENP2. For example, the phosphorylation levels of AKT are decreased in SENP2 over-expressing cells, while in MEF cells that SENP2 are knockouted, the levels of phosphorylated AKT (473S) protein are elevated. Furthermore, the LY294002 treatment rescued SENP2 deficiency induced metabolic defects. These data indicate that AKT phosphorylation is involved in glucose regulation by SENP2 both in MCF7 and MEF cells. The PI3K/AKT signaling pathway has been shown to increase glucose uptake by stimulating over-expression and membrane localization of GLUT1 in tumor cells [16]. This pathway also stimulates HK2 expression and translocalization to mitochondria. Consequently, the glucose phosphorylation is increased, as well as the expression of other glycolytic genes [17], [18], [19]. Stable depletion of HK2 has also been reported to inhibit aerobic glycolysis and promote normal oxidative glucose metabolism [20]. In our study, the expression of both Glut1 and HK2 are lower in SENP2 over-expression MCF7 cells than control cells. Consistently, their expression levels are induced in SENP2 knockout MEF cells. Therefore, suppression of glycolysis induced by over-expression of SENP2 could be partially mediated by decreased AKT phosphorylation, though we do not exclude that there may be other pathways which could also mediate the effect.

SUMO-specific protease 2 (SENP2) has a broad de-SUMOylation activity in vitro and in vivo [6]. *PTEN* is a tumor-suppressor gene that inhibits the PI3K/AKT/mTOR pathway by cleaving a phosphate group from the PI3K-activated second messenger PIP-3 [21]. Several studies have reported that PTEN can be sumoylated. Huang et al has reported that PTEN is covalently modified by SUMO1 at both K266 and K 254 sites in the C2 domain of PTEN [22]. González-Santamaría et al reported that PTEN is also post-translationally modified by SUMO1 and SUMO2 [23]. Therefore, we advance the hypothesis that SENP2 may regulate AKT phosphorylatio by controlling the activity of PTEN through de-sumoylation. In our system, whether SENP2 specifically de-SUMOylates PTEN, and inhibits its phosphotase activity to let the AKT over-activation, need to be explored in future.

Metabolic reprogramming is an essential hallmark of cancer cells, either as a consequence or as a cause [4]. Growing evidence showed that aerobic glycolysis contributes to cell proliferation. In our study, we found that SENP2 knockout MEF cells, exhibiting an increased aerobic glycolysis level, proliferate significantly faster than WT cells (Fig. S3. A). Consistently, MCF7-SENP2 cells, showing a reduced glycolysis level, tend to grow slower than MCF7-CON cells (Fig. S3. B). Moreover, we also found that SENP2 knockout MEF cells are more dependent on glucose for survival than WT cells (Fig. S3. C). Our data suggests a potential role of SENP2 in cell proliferation, which may be intertwined with an altered glucose metabolism.

A previous study from Agbor et al has reported that SUMO-1 promotes glycolysis in hypoxia [15]. Sumoylation is a dynamic process and is readily reversed by a family of SUMO-specific proteases (SENPs) [24]. Here, our study further find out that SENP2 can inhibit glycolysis both in MCF7 and MEF cells, which is consistent with the former results. Moreover, under normal condition, we find that MCF7 cells over-expressing SENP2 can reduce glucose uptake and lactate production while SENP2−/− MEF cells increase glucose uptake and lactate production. More than that, over-expressing SENP2 can also partially revert MCF7 cells from aerobic glycolysis to normal oxidative glucose metabolism. Finally, AKT phosphorylation (473S) is found significantly reduced in SENP2 over-expressed cells and consistently elevated in SENP2 knockout cells. PI3K/AKT inhibitor LY294002 can markedly rescue the phenotype induced by SENP2 deficiency. Therefore, the PI3K/AKT pathway is hypothesized to be essential for SENP2 regulating the glucose metabolism in MCF7 and MEF cells.

### Conclusions

Our study reports a negative role of SENP2 in the regulation of glycolysis. SENP2 over-expression in MCF7 breast cancer cells results in decreased glycolysis, while SENP2 knockout MEF cells show increased glycolysis. Furthermore, over-expression of SENP2 in MCF7 cells partially switches glucose from glycolysis to oxidative phosphorylation, which is a more effective way in using glucose. Our results further demonstrate that the PI3K/AKT pathway is critical for SENP2 mediated glucose metabolism. Our study reveals a novel function of SENP2 in the regulation of glucose metabolism, which may in part account for the molecular mechanisms of the Warburg effect.

## Supporting Information

Figure S1 **SENP2 represses glycolysis in a HIF1a-independent way.** (A) Western blotting of HIF1a in MCF7-CON and MCF7-SENP2 cells. (B) Glucose uptake and (C) Lactate production in MCF7-CON and MCF7-SENP2 cells under normal and Hypoxia condition. The data were presented as the mean ± SD of triplicate samples and normalized by cell number. *P<0.05.
(TIF)

Figure S2 **Fold change of glucose uptake after 0 uM, 10 uM and 20 uM LY294002 treatment for 48 h.** *P<0.05.
(TIF)

Figure S3 **SENP2 represses cell proliferation and SENP2-silenced MEF cells are addicted to glucose for survive.** (A) Growth curve of MEF-WT and MEF-SENP2−/− cells. (B) Growth curve of MCF7-CON and MCF7-SENP2 cells. (C) Growth curves of WT and SENP2−/− MEF cells in medium with glucose and without glucose. *P<0.05.
(TIF)

Table S1 **Real-time PCR primers used to amplify target genes.**
(DOC)
