# Supplementary figures and images for: Role of SUMO-Specific Protease 2 in Reprogramming Cellular Glucose Metabolism
Source: PLoS One. 2013 May 14;8(5):e63965. doi: 10.1371/journal.pone.0063965 (PMC3653847; doi:10.1371/journal.pone.0063965)

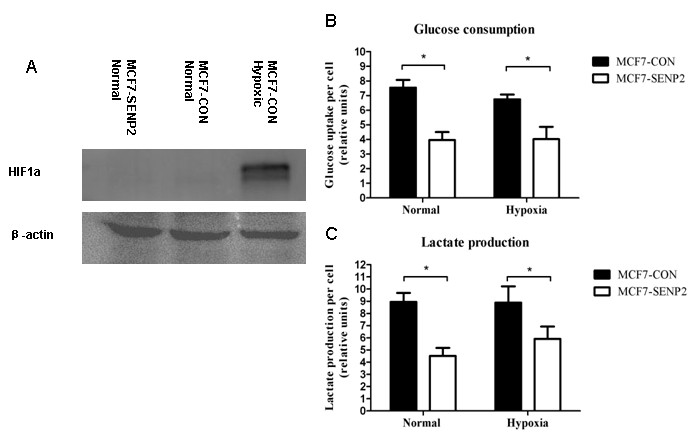

Supplement: Figure S1 — SENP2 represses glycolysis in a HIF1a-independent way. (A) Western blotting of HIF1a in MCF7-CON and MCF7-SENP2 cells. (B) Glucose uptake and (C) Lactate production in MCF7-CON and MCF7-SENP2 cells under normal and Hypoxia condition. The data were presented as the mean ± SD of triplicate samples and normalized by cell number. *P<0.05. (TIF) [file pone.0063965.s001.tif]

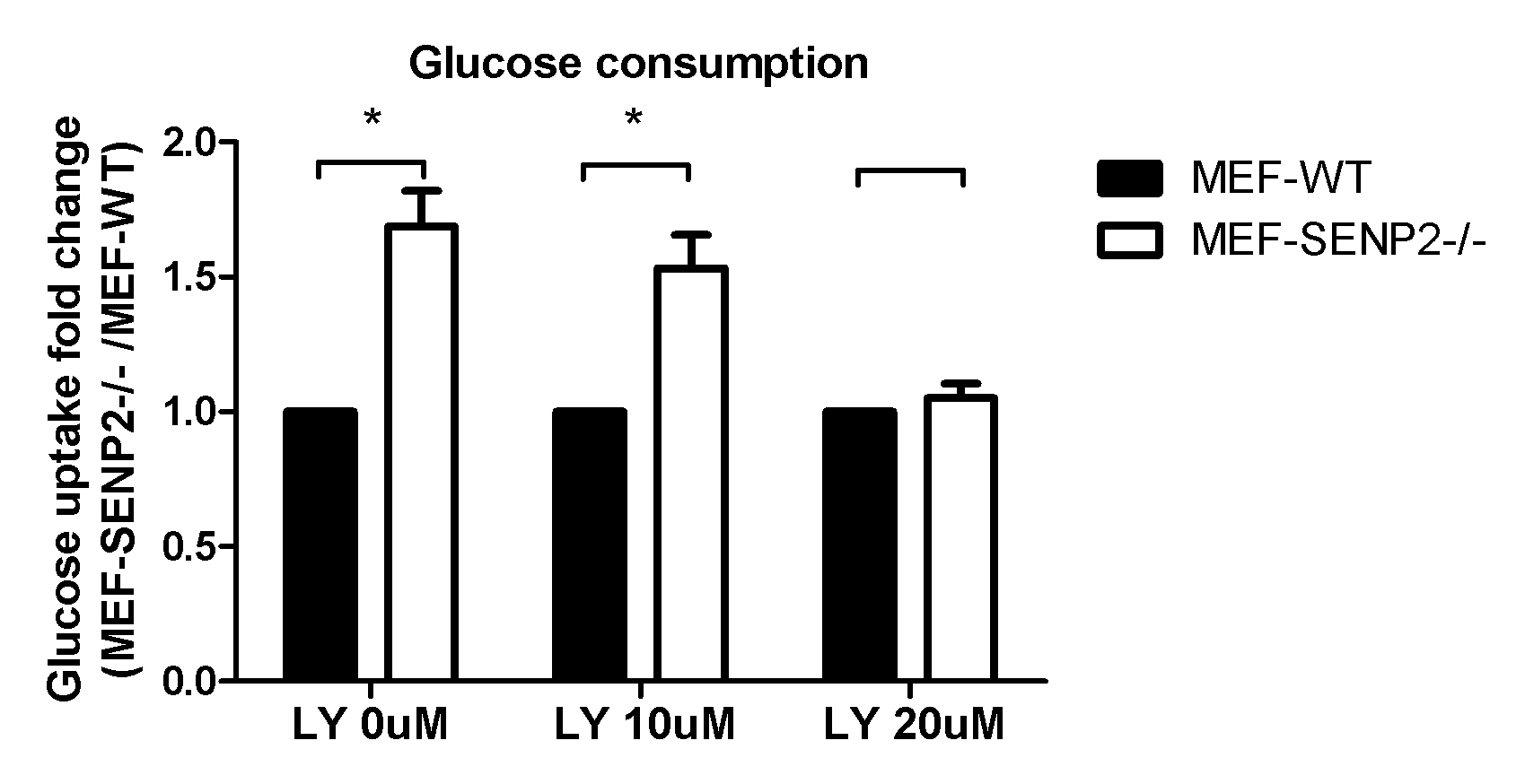

Supplement: Figure S2 — Fold change of glucose uptake after 0 uM, 10 uM and 20 uM LY294002 treatment for 48 h. *P<0.05. (TIF) [file pone.0063965.s002.tif]

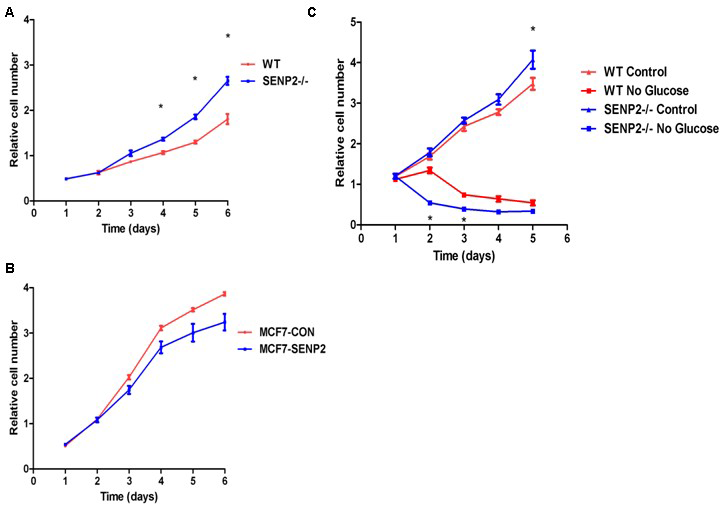

Supplement: Figure S3 — SENP2 represses cell proliferation and SENP2-silenced MEF cells are addicted to glucose for survive. (A) Growth curve of MEF-WT and MEF-SENP2−/− cells. (B) Growth curve of MCF7-CON and MCF7-SENP2 cells. (C) Growth curves of WT and SENP2−/− MEF cells in medium with glucose and without glucose. *P<0.05. (TIF) [file pone.0063965.s003.tif]
