# Supplementary material for: Role of SUMO-Specific Protease 2 in Reprogramming Cellular Glucose Metabolism
Source: PLoS One. 2013 May 14;8(5):e63965. doi: 10.1371/journal.pone.0063965 (PMC3653847; doi:10.1371/journal.pone.0063965)
Supplement: Table S1 — Real-time PCR primers used to amplify target genes. (DOC) [file pone.0063965.s004.doc]

Table S1. Real-time PCR primers used to amplify target genes

| Locus | Primers (5’–3’) |
| --- | --- |
| Glut1(Hm) | (F) ATCGTGGCCATCTTTGGCTTTGTG  (R)CTGGAAGCACATGCCCACAATGAA |
| HK2(Hm) | (F) TGAAGTTGGCCTCATTGTTGGCAC  (R) TTCTCCTTCCACCAGTTCCACGTT |
| PFK(Hm) | (F) TGGGAGCTTCGAGAACAACTGGAA  (R) ATTCAGGATGGCCAGGGAGAAGTT |
| ALDOA(Hm) | (F) GGCCATGCTTGCACTCAGAAGTTT  (R) AATGGCATTGAGGTTGATGGACGC |
| PGK1(Hm) | (F) TGGACAAGCTGGACGTTAAAGGGA  (R) AATTTGATGCTTGGGACAGCAGCC |
| PKM2(Hm) | (F) GTGCGAGCCTCAAGTCA  (R) ACGTGGGCGGTATCTGG |
| G6PD(Hm) | (F) AGAACATTCACGAGTCCTGC  (R) GTGGTCGATGCGGTAGATC |
| LDHA(Hm) | (F) GTGCACCCAGTTTCCACCATGATT  (R) TTCTTCAAACGGGCCTCTTCCTCA |
| PDK1(Hm) | (F) GGCTGGTTTTGGTTATGGATTG  (R) CTGGGAGTCTTTCTATTGAGTCTG |
| 18S(Hm) | (F) AGGCCCTGTAATTGGAATGAGTC  (R) GCTCCCAAGATCCAACTACGAG |
| SENP2(Hm) | (F) AGCCTGGTGGTGATTGACCTAAGA  (R) AGCTGTTGAGGAATCTCGTGTGGT |
| Glut1(Mus) | (F)TGTTGTAGAGCGAGCTGGACG  (R) GCCACGATGCTCAGATAGGACA |
| HK2(Mus) | (F) GTGCTCCGAGTAAGGGTGACAGAC  (R) CGGCAATGTGGTCAAACAGC |
| PFK(Mus) | (F) TGGACGAGGAGAGGTTTGACG  (R) TTCAGGATGGCCAGGGAGAAG |
| ALDOA(Mus) | (F) AAGTTATCAAGTCCAAGGGTGGTGT  (R) TCTTATACTGGGCACAGCGTTCAG |
| PGK1(Mus) | (F) GCCAAGTCCGTTGTCCTTATGAG  (R) TCAAGAACAGAACATCCTTGCCC |
| PKM(Mus) | (F) AAGTGTTTAGCAGCAGCTTTGATAG  (R) CGAGTCACGGCAATGATAGGAG |
| G6PD(Mus) | (F)TATTTATCATCATGGGTGCATCGG  (R) TGTGAGTCGTGAGCGGGCAT |
| LDHA(Mus) | (F) CGTTACCTGATGGGAGAGAGGCT  (R) GGCAACATTCACACCACTCCAC |
| PDK1(Mus) | (F) CATTAAAGATGGCTATGAGAACGCT  (R) TTGTCTGTCCTGGTGATTTCGC |
